# Transgenic *Xenopus laevis* Line for In Vivo Labeling of Nephrons within the Kidney

**DOI:** 10.3390/genes9040197

**Published:** 2018-04-06

**Authors:** Mark E. Corkins, Hannah L. Hanania, Vanja Krneta-Stankic, Bridget D. DeLay, Esther J. Pearl, Moonsup Lee, Hong Ji, Alan J. Davidson, Marko E. Horb, Rachel K. Miller

**Affiliations:** 1 Department of Pediatrics, Pediatric Research Center, UTHealth McGovern Medical School, Houston, TX 77030, USA; Mark.E.Corkins@uth.tmc.edu (M.E.C.); hlh2@rice.edu (H.L.H.); Vanja.Stankic@uth.tmc.edu (V.K.-S.); Bridget.D.Delay@uth.tmc.edu (B.D.D.); 2 Program in Biochemistry and Cell Biology, Rice University, Houston, TX 77005, USA; 3 Program in Genes and Development, MD Anderson Cancer Center UTHealth Graduate School of Biomedical Sciences, Houston, TX 77030, USA; moonsup.lee@nih.gov; 4 National Xenopus Resource and Eugene Bell Center for Regenerative Biology and Tissue Engineering, Marine Biological Laboratory, Woods Hole, MA 02543, USA; esther.pearl@kcl.ac.uk (E.J.P.); mhorb@mbl.edu (M.E.H.); 5 Department of Genetics, University of Texas MD Anderson Cancer Center, Houston, TX 77030, USA; hongji@mdanderson.org; 6 Department of Molecular Medicine and Pathology, University of Auckland, Auckland 1010, New Zealand; a.davidson@auckland.ac.nz; 7 Program in Genetics and Epigenetics, MD Anderson Cancer Center UTHealth Graduate School of Biomedical Sciences, Houston, TX 77030, USA; 8 Program in Biochemistry and Cell Biology, MD Anderson Cancer Center UTHealth Graduate School of Biomedical Sciences, Houston, TX 77030, USA

**Keywords:** *cdh17*, pronephros, mesonephros, kidney, nephron, live imaging, primary culture, *Xenopus*, transgenic

## Abstract

*Xenopus laevis* embryos are an established model for studying kidney development. The nephron structure and genetic pathways that regulate nephrogenesis are conserved between *Xenopus* and humans, allowing for the study of human disease-causing genes. *Xenopus* embryos are also amenable to large-scale screening, but studies of kidney disease-related genes have been impeded because assessment of kidney development has largely been limited to examining fixed embryos. To overcome this problem, we have generated a transgenic line that labels the kidney. We characterize this *cdh17*:eGFP line, showing green fluorescent protein (GFP) expression in the pronephric and mesonephric kidneys and colocalization with known kidney markers. We also demonstrate the feasibility of live imaging of embryonic kidney development and the use of *cdh17*:eGFP as a kidney marker for secretion assays. Additionally, we develop a new methodology to isolate and identify kidney cells for primary culture. We also use morpholino knockdown of essential kidney development genes to establish that GFP expression enables observation of phenotypes, previously only described in fixed embryos. Taken together, this transgenic line will enable primary kidney cell culture and live imaging of pronephric and mesonephric kidney development. It will also provide a simple means for high-throughput screening of putative human kidney disease-causing genes.

## 1. Introduction

*Xenopus laevis* is an established vertebrate model for studying developmental processes. *X. laevis* produce free-living, relatively transparent embryos, enabling direct visualization of development during chemical and genetic screens. This, combined with the numerous embryos produced in a single clutch, allows for hundreds of embryos to be manipulated and observed in a single experiment [1,2,3]. Additionally, the large size of the embryos and established fate maps allow for targeted microinjection into a selected blastomere to manipulate gene expression in a tissue of interest [4,5,6,7,8,9]. This method of targeted injections directs knockdown or overexpression of genes to a selected subset of tissues in F0 generation embryos. Targeted injections allow for a delivery of constructs to organs of interest, while avoiding tissues that affect early development and prevent assessment of subsequent phenotypes. This technique generates tissue-targeted knockdown or knockout mutant embryos similar to the Cre–LoxP systems commonly used in mouse and zebrafish studies.

Mammalian kidneys go through three developmental stages: the pronephros, mesonephros and metanephros [10]. Although the mammalian pronephros is non-functional, it is essential for the subsequent development of the mesonephric and metanephric kidney forms [11]. Each *X. laevis* pronephric kidney consists of one functional nephron which runs down the side of the tadpole. Like nephrons in the mammalian metanephros, the *X. laevis* pronephros consists of a glomus (functionally similar to the mammalian glomerulus), as well as proximal, intermediate, distal and connecting tubules, which together filter waste products and resorb nutrients from the blood [10,12,13,14]. Additionally, many of the markers that label each region of the kidney are conserved between the *Xenopus* pronephros and the mammalian metanephros [15,16].

*X. laevis* is a useful model for studying processes involved in vertebrate kidney development and disease [17]. The *X. laevis* pronephros undergoes rapid development and becomes functional within 2–3 days after fertilization [18,19]. A number of assays to assess kidney function, such as development of edema and visualization of the passage of fluorescent molecules through the kidney, have been developed for use in *X. laevis* [13,20]. Modulating the function or expression of putative disease-causing genes through the injection of morpholinos, messenger RNA (mRNA) or clustered regularly interspaced short palindromic repeats (CRISPR)–Cas9 gene editing constructs results in kidney developmental phenotypes in *X. laevis* [21,22]. This allows for further study of the role that these genes play in human kidney pathologies.

*X. laevis* is also a useful model for use in stem cell and primary cell culture research. In early embryos, cells are weakly held together and can be easily dissociated in calcium- and magnesium-free saline. An established example of this is the animal cap [23]. Additionally, the cells of *X. laevis* embryos contain yolk, allowing them to survive in simple saline solutions for days. *X. laevis* cells can also survive at temperatures from 15 to 25 °C under normal atmosphere, allowing for easy growth without the need for a special incubator. Given that there are no clear live markers for kidney cells, one limitation of primary tissue culture of kidney cells in *X. laevis* is the inability in live cells to identify cells of interest.

Although the external development of *X. laevis* embryos allows for live imaging of many tissues, [24] live imaging of the kidney is difficult because there are currently no live markers. To facilitate the study of the dynamic cellular movements that occur during nephrogenesis, we generated a *X. laevis* transgenic line that labels the kidney cells. Here, we characterized this *cdh17*:eGFP line and demonstrate its usefulness in examining kidney development in vivo over a wide range of developmental stages. We also show that GFP expression within the labeled kidney is useful for monitoring the effects of targeted microinjection of morpholinos. Additionally, we develop a method for primary culture of *X. laevis* kidney cells. This transgenic line allows for live embryonic kidney labeling without the need to fix and stain embryos, enabling live observation of *X. laevis* kidneys and kidney function during development.

## 2. Materials and Methods

### 2.1. Transgenic Animal Generation and Embryo Culture

**Figure 1:**
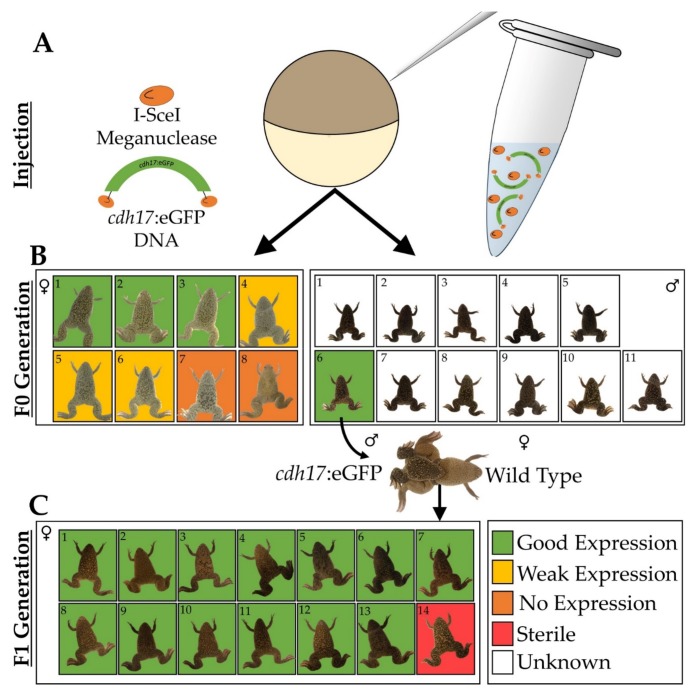
Schematic of *cdh17*:eGFP transgenic line creation. (**A**) *cdh17*:eGFP construct flanked by I-SceI sites was co-injected with I-SceI meganuclease into one-cell embryos. (**B**) Eight female and 11 male *Xenopus laevis* adults were grown from these F0 embryos. (**C**) Upon mating of an F0 transgenic male to a wild type female, 14 F1 females were generated.

The *cdh17:*eGFP [Xla.Tg(Dre.*cdh17*:eGFP)^NXR^; RRID:NXR_0.0102] transgenic frog line was generated by I-SceI mediated integration [25,26]. Approximately a 4.3-kb genomic fragment upstream of the *Danio rerio cdh17* gene was amplified from pTol2(*cdh17*::eGFP) [27,28] and cloned into the I-SceIpBSIISK+ vector. GFP-positive transgenic embryos were generated and maintained until sexual maturity at the National Xenopus Resource (Woods Hole, MA, USA). Transgenic F0 males were crossed to wild type *X. laevis* females to generate backcrossed F1 generation animals (Figure 1).

Wild type *X. laevis* adult males were purchased from Nasco (LM00713M, Fort Atkinson, WI, USA). Embryos were obtained from *cdh17*:eGFP adult females and reared as previously described [29]. All embryos were grown in 1/3× Marc’s Modified Ringer’s (MMR) (33 mM NaCl, 0.66 mM KCl, 0.33 mM MgSO_4_, 0.66 mM CaCl_2_, 1.66 mM HEPES pH 7.4) [29]. This protocol was approved by the UTHealth’s Center for Laboratory Animal Medicine Animal Welfare Committee, which serves as the Institutional Animal Care and Use Committee (protocol #: AWC-16-0111).

### 2.2. Immunostaining

Embryos were staged [19], fixed [9], and immunostained using established protocols [30]. Proximal tubule lumens were labeled with antibody 3G8 (1:30, European Xenopus Resource Centre, Portsmouth, United Kingdom), while cell membranes of the intermediate, distal and connecting tubules were labeled with antibody 4A6 (1:5, European Xenopus Resource Centre) [31]. Proximal tubule lumens were also detected using Fluorescein isothiocyanate (FITC)-conjugated lectin from *Erythrina cristagalli* (1:1000, FL-1141 Vector labs, Burlingame, CA, USA). Rabbit anti green fluorescent protein (anti-GFP) (1:500, iclLab, Portland, OR, USA) antibody was used to detect GFP expression. Goat anti-mouse or anti-rabbit conjugated to Alexa Fluor 488, Alexa Fluor 555, or Alexa Fluor 647 (1:2000, Invitrogen, Carlsbad, CA, USA) secondary antibodies were used to visualize antibody staining.

### 2.3. Imaging

Embryos were scored and imaged using an Olympus SZX16 fluorescent stereomicroscope (Olympus, Tokyo, Japan) equipped with an Olympus DP71 camera. Confocal kidney images were taken using a Zeiss LSM800 confocal microscope (Zeiss, Oberkochen, Germany). Fixed embryos were cleared in a solution of BABB/Murray’s clearing solution (1:2 volume of benzyl alcohol to benzyl benzoate). For live imaging, animals were anesthetized with 10 μg/mL Benzocaine in 1/3× MMR [32,33]. For time-lapse movies, embryos were immobilized using coverslips anchored down using vacuum grease [34]. An image was taken every 30 min over a period of 15 h. Images were processed with Zen blue (Zeiss), Adobe Photoshop, and Microsoft PowerPoint.

### 2.4. Secretion Assay

Two–4 pulses of 5 nL of 1 mg/mL rhodamine-dextran 10,000 MW (10–20 nL total) was injected into the coelomic cavity above the heart of benzocaine-anesthetized stage 40–43 embryos [13]. Embryos were either directly visualized with a dissection microscope, or placed on a confocal microscope for high resolution imaging.

### 2.5. Kidney Cell Primary Culture

Animals expressing GFP within the kidney were identified by fluorescent microscopy. The pronephric primordium was identified by anatomical position and explanted using sharp forceps from stage 30–32 embryos under white light. All explants were carried out on 2% agar-coated plates. Approximately 10–20 kidney explants were disassociated in Calcium Magnesium Free Media (CMFM) [1/3× MMR without MgSO_4_ and CaCl] for 1 h. Once dissociated cells were washed in Danilchik’s for Amy (DFA) [53 mM NaCl, 5 mM Na_2_CO_3_, 4.5 mM potassium gluconate, 32 mM sodium gluconate, 1 mM CaCl_2_, 1 mM MgSO_4_, 0.1% (*w*/*v*) BSA, buffered to pH 8.3 with 1 M bicine] and allowed to attach. Dissociation and attachment procedures were carried out in Attofluor™ Cell Chamber (Thermo Fisher A7816, Waltham, MA, USA) with fibronectin-coated coverslips (Roche 10838039001, Basel, Switzerland). Alternatively, to increase the yields of individualized cells, explants were treated with Trypsin-EDTA (Ethylenediaminetetraacetic acid) (Sigma T4049, St. Louis, MO, USA) for 5 min instead of CMFM, followed by inactivation of Trypsin-EDTA with 10% fetal calf serum in DFA.

### 2.6. Microinjection

Microinjections were performed as previously described [9]. 10 nL of injection mix (described below) was injected into the indicated blastomere. 20 ng of Daam1 morpholino 5′-GCCGCAGGTCTG TCAGTTGCTTCTA-3′ or Standard morpholino 5′-CCTCTTACCTCAGTTACAATTTATA-3′ were injected along with 25 ng rhodamine-dextran as a tracer, to mark targeted cells, into the V2 blastomere at the 8-cell stage to target the kidney [6,35]. Error is given as Standard Error of the Mean (SEM) across at least three trials.

## 3. Results

### 3.1. Generation of Xenopus laevis cdh17:eGFP Line

In zebrafish (*Danio rerio*), Cadherin-17 (*cdh17*) is a gene that functions in maintaining pronephric integrity [36]. Previously, the *cdh17* promoter was used to generate a zebrafish transgenic line to label the pronephric and the mesonephric kidney [27,28]. Given that this promoter was previously characterized in zebrafish, we confirmed pTol2(*cdh17*::eGFP) expression in the *X. laevis* pronephros in F0 embryos using the Tol2 system [37]. To improve the transgene integration rate into the *X. laevis* genome, we cloned the zebrafish *cdh17*::eGFP fragment into the I-SceIpBSIISK+ vector to generate a *X. laevis* transgenic line [27]. This vector allows for I-SceI-mediated rather than Tol2-mediated integration. Eight F0 GFP-positive female animals and 11 F0 males were grown until adulthood. A GFP-positive male was mated to a wild type female to generate 14 F1 generation female animals (Figure 1). Of the eight F0 females, six animals produced *cdh17*:eGFP-positive offspring, although three of the F0 females produced offspring with weak GFP expression. The remaining two F0 animals produced embryos with undetectable levels of GFP in the kidney. Thirteen of the F1 animals produced uniformly strong kidney GFP expression, while one F1 female was sterile and did not produce any eggs. The consistency of GFP expression observed in the embryos of the fertile F1 females was anticipated given that these females were produced from the same transgenic father and wild type mother. In contrast, the F0 females produced progeny with a range of GFP intensity, likely due to the different integration events in each animal. 

### 3.2. cdh17:eGFP Colocalizes with Kidney Markers

**Figure 2:**
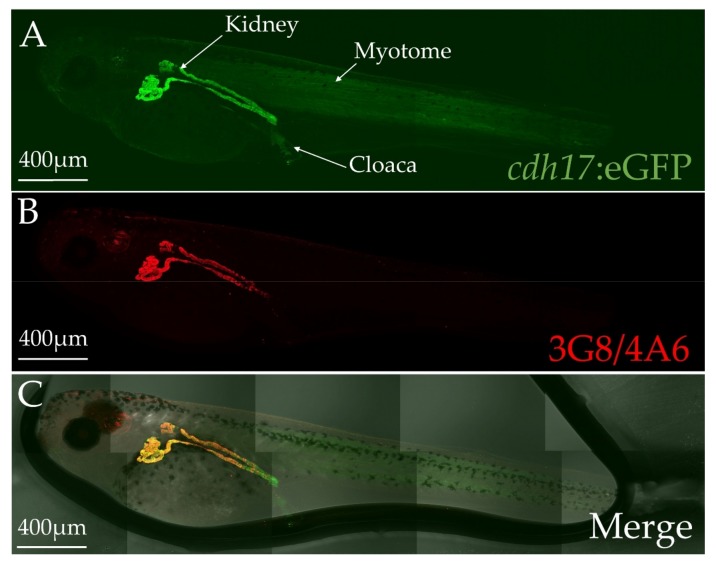
*cdh17*:eGFP expression co-localizes with the kidney markers 3G8 and 4A6. Stage 42 *cdh17*:eGFP transgenic *X. laevis* embryos were co-immunostained with antibodies for green fluorescent protein (GFP) and the kidney markers 3G8 and 4A6. (**A**) GFP immunostained *cdh17*:eGFP embryo. (**B**) 3G8/4A6 immunostained embryo. (**C**) Merged differential interference contrast (DIC) and fluorescent image of *cdh17*:eGFP embryo. Embryo is surrounded in a bubble of 1:2 volume benzyl alcohol to benzyl benzoate (BABB).

**Figure 3:**
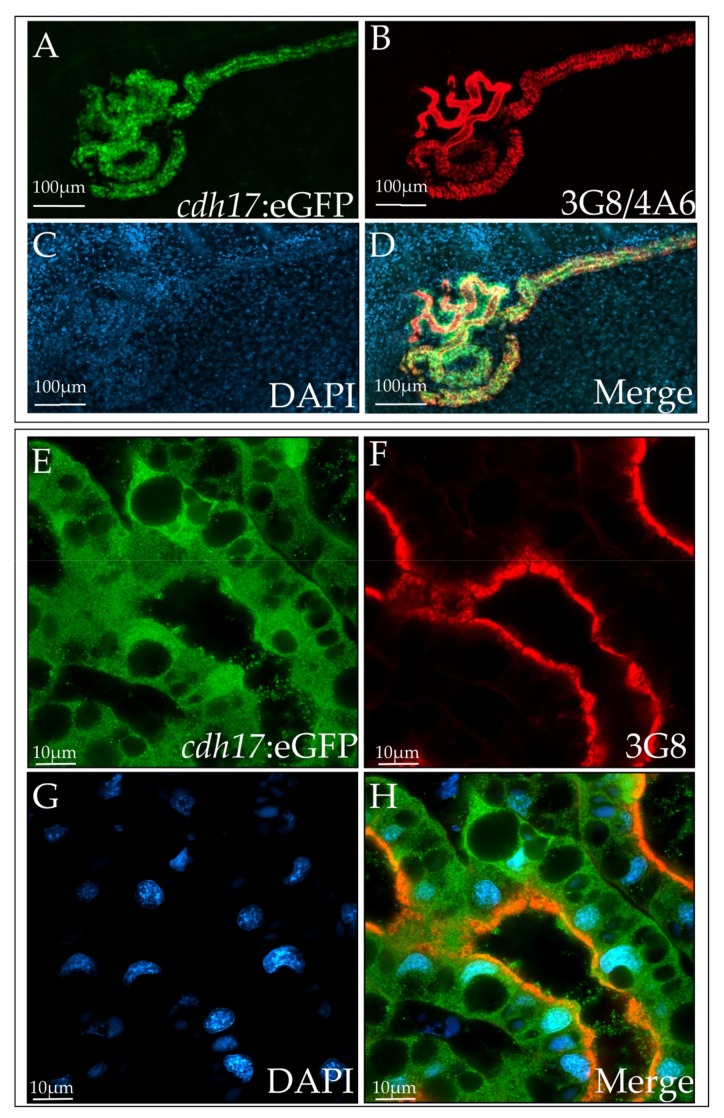
Subcellular GFP expression in the *cdh17*:eGFP line is largely cytoplasmic and nuclear. Fixed stage 42 *cdh17*:eGFP embryos were stained with (**A**,**E**) anti-GFP, (**B**,**F**) 3G8/4A6, and (**C**,**G**) DAPI (4′,6-diamidino-2-phenylindole). (**E**–**H**) Magnified image of proximal tubules.

**Figure 4:**
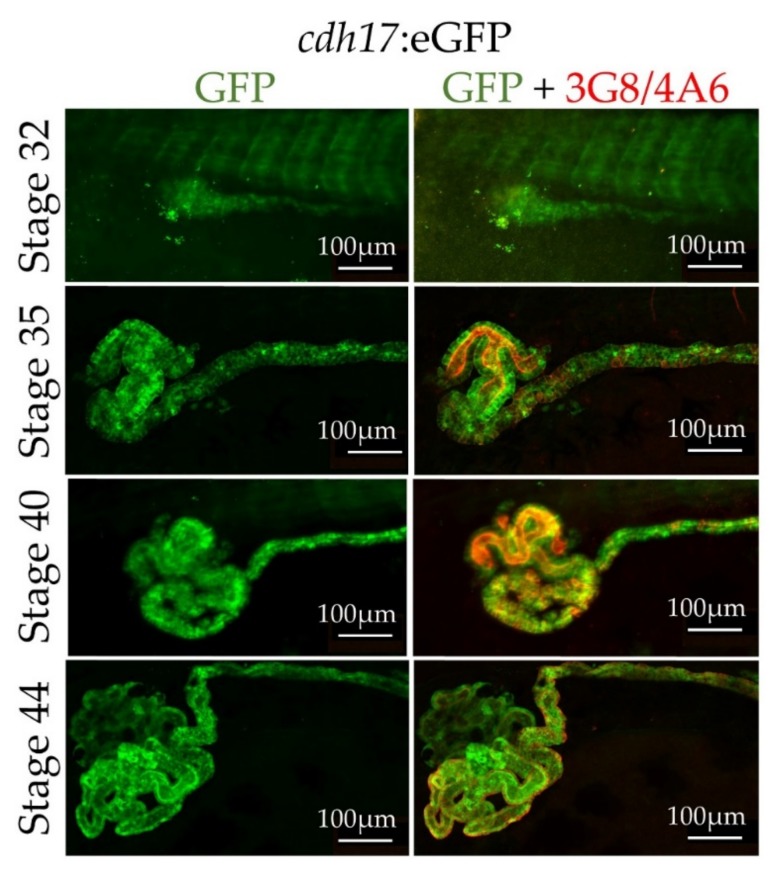
*cdh17*:eGFP labels the kidney throughout pronephric development. Stage 32 through stage 44, *cdh17*:eGFP transgenic *X. laevis* embryos were immunostained with antibodies for GFP and the kidney markers 3G8 and 4A6. GFP expression is present throughout pronephric kidney development. (**Left**) GFP expression, (**right**) GFP (Green) and 3G8/4A6 (red) kidney marker expression.

To assess the spatial expression of GFP in F2 embryos, we grew tadpoles to stage 40, fixed, and then stained them with markers of the pronephros. The 3G8 antibody labels the lumen of the proximal tubules, while the 4A6 antibody labels the cell membranes of the intermediate, distal and collecting tubules (Figure 2, Figure 3 and Figure 4) [31]. Additionally, we used Lectin as a marker to further verify proximal tubules (Video S1) [35]. GFP is expressed in the same cells as these markers, indicating that the kidney cells are expressing GFP. Additionally, a few tissues other than the kidney weakly expressed GFP, including the myotome and a population of cells dispersed within the cloaca (Figure 2). However, in tadpole stages, this expression is weak in comparison with the kidney expression. As predicted, GFP expression appears to be cytoplasmic and nuclear, since a signal peptide was not included in the construct (Figure 3).

We find that the *cdh17*:eGFP transgene is not deleterious to the embryos. The 3G8/4A6 staining in the transgenic embryos looks similar to their non-transgenic siblings as well as wild type embryos grown in parallel, suggesting that the transgene does not alter kidney morphology. Additionally, the transgenic animals do not develop edema, indicating that they have functional kidneys. We also see that 52% ± 7% (*n* = 488 embryos) of the embryos had GFP expression in their kidneys. This is about the predicted rate, given that the transgenic mothers were heterozygous for the transgene, and the testes used to fertilize the eggs were from wild type *X. laevis* males. We also find that the transgenic embryos grow at the same rate as their wild type siblings. Together, our data suggest that the *cdh17*:eGFP transgene is not being selected against and is likely not affecting embryo survival or kidney morphology.

### 3.3. In Vivo Characterization of cdh17:eGFP Transgenic Line

**Figure 5:**
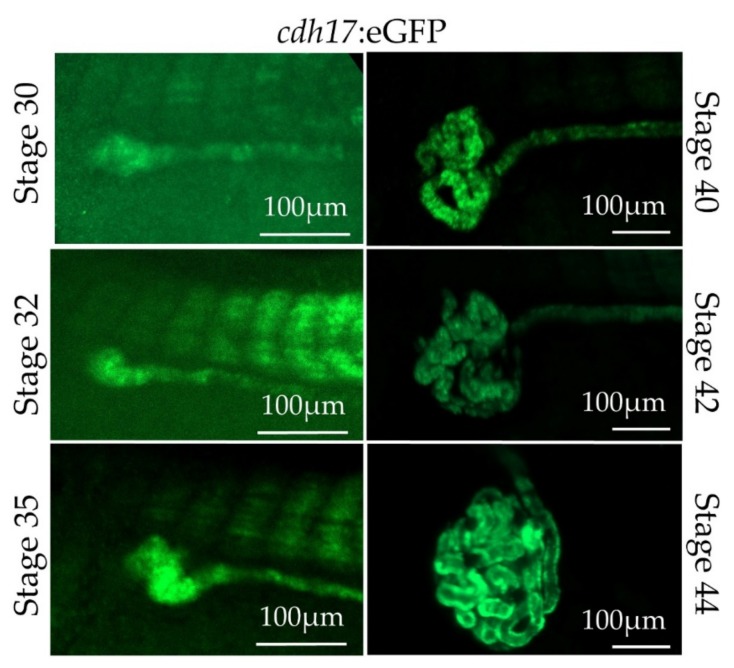
Live imaging of kidney development using *cdh17*:eGFP reporter line. Kidney development within a transgenic embryo was visualized by GFP expression from stages 30 to 44.

As there is still much to be learned about how the kidney tubules normally develop, we wished to observe nephrogenesis in real time. To test the feasibility of live imaging of the *cdh17*:eGFP line, we anesthetized embryos with benzocaine, immobilized them using coverslips, and then directly imaged them (Figure 5). *cdh17*:eGFP is expressed in the developing pronephros between tadpole stages 30 through 44. We also imaged embryos starting at stage 35 over a period of 15 h (Video S2). We were able to revive animals by washing them following benzocaine treatment and imaging, indicating that embryos were not severely harmed in the process. 

### 3.4. Assessment of Kidney Function in cdh17:eGFP Embryos

**Figure 6:**
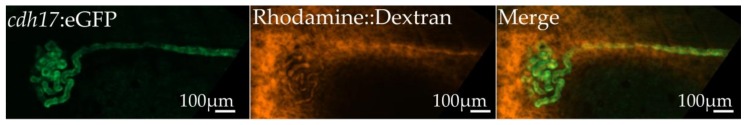
*cdh17*:eGFP transgenic line labels the kidney for tracking of rhodamine secretion. Rhodamine-dextran was injected into the coelomic cavity of stage 42 *X. laevis* embryos and tracked using GFP as a marker for the kidney.

Various dyes, such as 10,000 MW dextrans, are secreted into the urine by the embryonic *Xenopus* kidney, allowing for a simple visual readout of kidney function. This is performed by injecting labeled substances into the coelomic cavity. Uptake of the dye into the kidney can be directly visualized, followed by excretion of the dye in the urine. It takes approximately five minutes for the dyes to be secreted and visualized in real time in live embryos, allowing for easy phenotypic determination of kidney functionality (Video S3). However, coelomic dye injection can result in a strong signal from the coelomic cavity, making the tubules of the kidney difficult to visualize. The contrast between the GFP-labeled kidney tubules and the red rhodamine-dextran dye *in the cdh17*:eGFP line, facilitates the visualization of the tubules and the localization of the dextran passing through them (Figure 6).

### 3.5. Primary Culture of Xenopus laevis Kidney Cells

**Figure 7:**
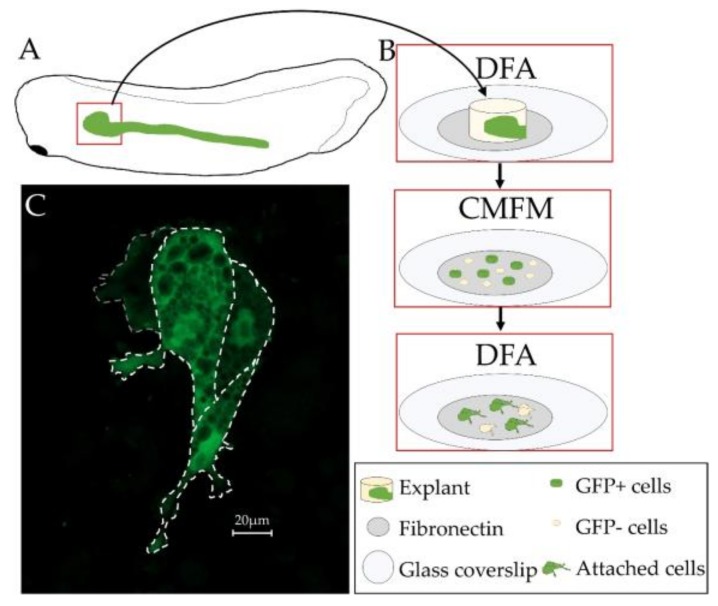
*X. laevis cdh17*:eGFP transgenic line is useful for primary culture of kidney tubules. (**A**) Diagram showing region of embryo that was explanted. (**B**) Schematic of methodology used to culture cells. Media used for each step is as indicated DFA (Danilchik**’**s for Amy) and CMFM (Calcium Magnesium Free Media). (**C**) Image of GFP-positive cells cultured for 24 h in DFA. Cells are outlined using dashed lines.

*X. laevis* embryos are amenable to dissection and primary cell culture. However, primary culture of *X. laevis* kidney cells has not been previously performed. Given that the *cdh17*:eGFP expression in the kidney allows for easy tracking of the dissociated cells, we developed a protocol for culturing kidney cells on coverslips. Similar to animal caps, the cells of the kidney disassociate in calcium- and magnesium-free media. After disassociation, cells were plated on fibronectin coated coverslips. The cells attached to the coverslips in a few hours, and were viable for at least 24 h. Kidney cells were easily detectable by their GFP expression (Figure 7). This 24 h point was chosen as it would be long enough to assess if our treatment of the cells was acutely toxic, or if the GFP expression reduced following explant of the cells without having to use media containing growth factors. GFP expression did not appear to reduce in strength over the 24 h culture period. The vast majority of explanted cells appeared healthy by GFP expression. As single cell suspensions were difficult to obtain with this method, we also utilized Trypsin-EDTA to completely disassociate the cells. We found that the cells were still viable after treatment, though many cells were lost during the washing steps.

### 3.6. Expression of Green Fluorescent Protein in Adult Animals

**Figure 8:**
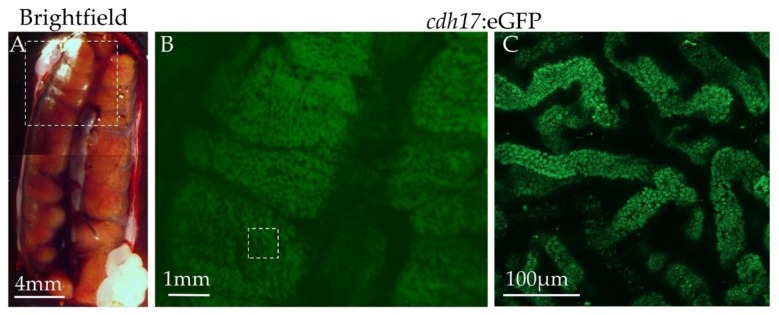
*X. laevis* adult mesonephric tubules express *cdh17*:eGFP. Adult kidneys were dissected, then imaged from an F1 generation *cdh17*:eGFP animal. (**A**) Brightfield image of *X. laevis cdh17*:eGFP kidney. (**B**,**C**) GFP fluorescence imaging of *cdh17*:eGFP kidney. Dashed boxes indicate regions taken for fluorescence imaging.

In zebrafish, the *cdh17*::eGFP reporter line labels the pronephric as well as the mesonephric kidneys [28]. To test weather this holds true in *X. laevis*, we euthanized two F0 and one F1 generation adult female animals. All of these frogs tested positive for GFP expression within the kidney as tadpoles, though the two F0 generation animals did not produce GFP-positive offspring, and the F1 generation animal was sterile. We imaged the mesonephric kidneys and found that the *cdh17*:eGFP reporter is expressed in tubules within the mesonephric kidney (Figure 8). To ensure that the signal we observed was not due to background signal, an adult wild type animal kidney was imaged and no detectable green fluorescent signal was observed.

### 3.7. Utility of cdh17:eGFP for Identifying Genes Involved in Kidney Diseases and Development

We next tested the utility of the *cdh17*:eGFP line to determine genes that result in kidney malformations. In *X. laevis,* antisense morpholinos are commonly used to alter gene activity [38]. As many genes that play a role in kidney development also play a role in the development of other tissues, we microinjected the V2 blastomere to target the kidney progenitors while avoiding most other tissues [9]. To test the potential of utilizing the *cdh17*:eGFP transgenic line to identify genes involved in kidney development, we chose to assess the expression of the *cdh17*:eGFP reporter upon disruption of Daam1.

**Figure 9:**
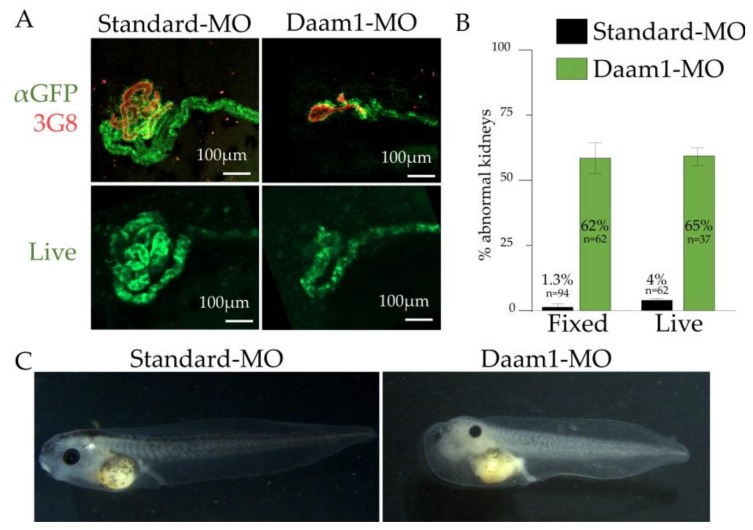
Disruption of kidney development using Daam1 morpholino (MO) can be tracked by *cdh17*:eGFP. *cdh17*:eGFP transgenic embryos were injected with Standard or Daam1 morpholino, then stage 42 embryos were assayed for kidney abnormalities. (**A**) Representative images of kidneys under indicated conditions. (**B**) Percentage of abnormal kidneys under indicated conditions. (**C**) Daam1 morpholino was injected into both V2 blastomeres, resulting in the development of edema, which indicates kidney functional defects. Arrow indicates site of edema.

Daam1 is a component of the planar cell polarity pathway, and loss of Daam1 is known to disrupt tubule morphology [35]. We injected Daam1 or a standard control morpholino into the V2 cell of 8-cell stage *cdh17*:eGFP transgenic embryos to target the kidney (Figure 9). The embryos were then fixed and immunostained with αGFP and 3G8/4A6 antibodies, allowing us to compare the effectiveness of monitoring GFP expression versus validated kidney markers to assess kidney development [29]. By monitoring the phenotype using GFP expression as a marker for kidney tubules, we were able to clearly visualize the effects of the morpholino injections on kidney development on the injected side of the embryos as compared with the uninjected side. Of the 62 transgenic embryos injected with Daam1 morpholino, 62% showed disrupted kidney tubule development, while only 1.4% of the 94 embryos injected with standard morpholino showed disrupted kidney development. In order to verify that our Daam1 morpholino was affecting kidney function as well as morphology, we assayed embryos for the development of edema after knocking down Daam1 in both kidneys: we found that they do develop edema.

The major advantage of the *cdh17*:eGFP animals is that kidney development can be directly visualized in living embryos. This reduces the time, effort, and cost needed to screen for disease-causing genes. In order to directly compare the efficacy of using living embryos, we injected Daam1 or standard morpholino and then assayed for abnormal GFP expression (Figure 9). Although the signal intensity is weaker as compared to the immunostained embryos, we find that immunostained kidneys show a similar number of abnormal kidneys when compared to living embryos. The added benefit of not needing to stain the embryos prior to imaging saves approximately three days of processing time, allowing for faster screening of embryos for phenotypes, and making this transgenic line suitable for high throughput screening of human kidney development genes.

## 4. Discussion

*X. laevis* is an attractive model for identifying genes responsible for human diseases [39]. Approximately 79% of genes implicated in human diseases have *X. laevis* orthologues, including genes that are involved in human kidney disorders [40]. Many of these disease-causing genes display phenotypes in the *X. laevis* pronephric kidney [21], although the mechanisms by which these disease-causing genes affect kidneys remain unknown. The *cdh17*:eGFP line allows us to follow the developmental processes underlying kidney formation as they are occurring, in order to further understand how kidney defects arise during embryonic development. The rapid development and large numbers of embryos allows for in vivo and real-time phenotypic assessment of large numbers of genes in a considerably shorter time than would be possible using mammalian systems.

There is still much to be learned about normal kidney development. This line will allow us to follow morphogenetic movements within the pronephric and mesonephric kidney throughout development. The *cdh17* promoter drives GFP expression beginning around embryonic stage 30 (early tadpole stage). This is before the 3G8 and 4A6 antibodies label the pronephros and during a time when the proximal tubules undergo large developmental changes. Additionally, the *cdh17*:eGFP reporter line labels the pronephric as well as the mesonephric kidneys of *X. laevis*. Little is known about the pronephric to mesonephric transition, and with this line, we have the potential to monitor these developmental changes as they occur.

Examining cells in culture provides some advantages when performing certain experiments. The primary advantage is that it is easier to visualize the staining of subcellular components in cells in culture. In the whole embryo, visualization of through other tissues may decrease image quality by blocking and refracting light. Additionally, getting antibodies to penetrate deeply into the embryo requires chemicals such as methanol or Proteinase K, which may be incompatible with some antibodies or stains. Additionally, primary cell culture has the advantage that cells can be isolated from external sources of growth factors. This allows for easier studying of factors that function in specializing cells into tissues of interest.

Previously, an immortal kidney cell line (A6) was generated from spontaneous kidney tumor in *X. laevis* [41]. Although stable transgenic cell lines have been generated using A6 cells, this process is frequently difficult, because A6 cells grow slowly and transfection rates are low [42,43,44]. Additionally, these cells are a cancerous line that requires growth factors to survive [43]. Here we describe a novel method of isolating, culturing and identifying primary cell lines using *X. laevis*. Primary culture of these kidney cells has advantages over traditional cell culture. Primarily, these non-cancerous cells allow for better modeling of normal kidney functions. As these cells contain their own yolk, they can be grown in a saline solution without animal serum. This eliminates many factors that may induce these cells to take on abnormal characteristics. Additionally, as injections are routine, transfection of these cells are not necessary. Instead, embryos can be injected with morpholinos, RNAs or CRISPR constructs, and then the affected kidney cells can be cultured and assayed. GFP expression allows for confirmation that the cells are kidney cells, and also allows for Fluorescence activated cell sorting (FACS) analysis of GFP-positive cells, a technique that would not have been feasible before this line was developed.

We show that GFP is expressed under faithful control of the *cdh17* promoter in the kidneys during organogenesis, and that this expression co-localizes with known kidney markers. We have used morpholino knockdown of Daam1 to confirm that the GFP expression in the kidney recapitulates phenotypes described previously in fixed embryos. We also demonstrate that live imaging of kidney development is feasible with this line, and that GFP expression functions as a reliable marker of kidney cells in secretion assays. Additionally, we have also developed a novel method for culturing primary cell lines from *X. laevis* kidneys. Taken together, this transgenic *X. laevis* line will be a useful tool, enabling high throughput screening of putative human kidney disease-causing genes.

## Supplementary Materials

The following are available online at http://www.mdpi.com/2073-4425/9/4/197/s1, Video S1: 3D structure of stage 40 cdh17:eGFP X. laevis. Transgene expression labeled using anti-GFP antibody (green), and Erythrina cristagalli lectin labeling the proximal tubules (red), Video S2: Live imaging of kidney development starting at stage 38 over a period of 15 h, Video S3: Movie of secretion assay in stage 42 X. laevis embryo.
